# The Impact of COVID-19 on Reconstructive Surgery for Burns and Complex Wounds

**DOI:** 10.1177/22925503221094104

**Published:** 2022-05-11

**Authors:** Alan D. Rogers

**Affiliations:** 1 483372 Ross Tilley Burn Centre, Sunnybrook Health Science Centre, Toronto, Ontario, Canada; 2 Division of Plastic and Reconstructive Surgery, University of Toronto, Toronto, Ontario, Canada

To the Editor

Much has been written of the considerable backlog of surgery resulting from the COVID-19 pandemic^1–3^ and its impact has not spared any surgical speciality.^4–5^ Of the areas within the scope of practice of burn centres, minimal attention has been placed on complex wound care and burn reconstruction in comparison with acute burn care.^6–8^

The number of cases performed at a verified regional burn centre in Canada during the last 2 years (since the pandemic was declared) was evaluated, and compared to the three preceding years. The 5 years under consideration began on March 1, 2017 and concluded on February 28, 2022. Cases were categorized into 3 groups: “Acute burn surgery” was distinguished from “burn reconstruction”, which refers to releases of burn scar contractures or scar revisions, while “complex wound surgery” is for, *inter alia,* pressure injuries, necrotizing soft tissue infections (NSTI), and extensive soft tissue trauma.

The mean number of acute burn surgeries per year during the pandemic was similar to the number per year preceding the pandemic (Table 1); the mean number of all cases performed per burn surgeon per year was 99 (range 79-128.5), down from 110.3 (range 76-155) (Table 2). While the mean case duration was longer during the pandemic, the burn operating room occupancy was not significantly different from prior years (Table 3).

**Table 1.** Surgical Cases Undertaken by the Burn Surgery Service, March 1, 2017 until February 28, 2022, divided into categories.

	2017^a^	2018^b^	2019	Mean before pandemic	2020	2021	Mean during pandemic
Acute burn surgery	306	289	328	308	318	317	317.5
Burn reconstruction	58	68	50	58.7	33	30	31.5
Complex wound surgery	58	116	59	77.7	51	25	38
Total cases per year	422	473	437	444	410	372	391

Note: ^a^2017 = March 1, 2017 to February 28, 2018.

^b^ 2018 = March 1, 2018 to February 29, 2019.

**Table 2.** Surgical Cases, Divided by the 4 Burn Surgeons, Before and During the Pandemic.

Surgeons	2017	2018	2019	Mean before pandemic	2020	2021	Mean during pandemic
#1	124	198	143	155	138	119	128.5
#2	130	96	122	116	112	105	108.5
#3	96	93	93	94	83	75	79
#4	71	85	72	76	86^a^	74^a^	80^a^
Mean per surgeon	105.3	118	107.5	110.3	104.8	93.3	99

Note: ^a^#4 = Two surgeons shared this resource allocation during 2020 and 2021.

**Table 3.** Operating Room use by the Burn Service Prior to and During the Pandemic.

	2017	2018	2019	Prior to pandemic	2020	2021	During pandemic
Total cases per year	422	473	437	444	410	372	391
Mean time per case (minutes)	175.4	169.9	183.6	176.3	194.3	191.3	192.8
Total operating time per year (hours)	1231	1336	1337	1301	1328	1186	1257

There was a significant reduction in the number of burn reconstructions undertaken during the pandemic when compared with the pre-pandemic period, with a mean of 31.5 cases performed per year, in comparison with a mean of 58.7 pre-pandemic; the deficit approximates the mean total number of burn reconstructions usually performed per year (n = 58) (Table 1).

Furthermore, there was a significant reduction in the number of complex wound cases during the period; the low number performed during 2021 (n = 25) was responsible for this difference. As was the case with burn reconstruction, the total number of cases “not performed” during the pandemic (n = 76), was similar to 1 year of these cases (n = 77.7) (Table 1).

The reduction in complex wound and burn reconstruction surgeries reflects policies to protect the specialist nursing workforce at the regional burn centre. Our group and others have previously demonstrated the impact on outpatient care, admissions and acute surgical resources, and described initiatives to replace in-person with virtual consultation whenever possible.^7,9,10^ For vulnerable patient populations with suboptimal access to surgical care, the pandemic has resulted in exacerbation of pressure injuries, and delays in definitive care for NSTI, and care may have been provided in environments unfamiliar or ill-equipped to provide the kind of holistic management available at a multidisciplinary burn centre.^11–13^ Reduced opportunities for trainees to participate in surgery for complex wounds and scars have also been evident, and may further entrench a system favouring sub-speciality over general plastic surgery practice.

In conclusion, this work highlights that while acute burn surgery has continued without significant impact at this regional burn centre, this may have been possible only at the expense of the delivery of care to other patient populations; it remains to be seen how this deficit can most effectively be addressed.
